# Nucleosomes undergo slow spontaneous gaping

**DOI:** 10.1093/nar/gkv276

**Published:** 2015-03-30

**Authors:** Thuy T.M. Ngo, Taekjip Ha

**Affiliations:** 1 Center for Biophysics and Computational Biology, University of Illinois at Urbana-Champaign, Urbana, IL 61801-2902, USA; 2 Department of Physics, Center for Physics in Living Cells, University of Illinois at Urbana-Champaign, Urbana, IL 61801-2902, USA; 3 Carl R. Woese Institute for Genomic Biology, University of Illinois at Urbana-Champaign, Urbana, IL 61801-2902, USA; 4 Howard Hughes Medical Institute, University of Illinois, Urbana, IL 61801-2902, USA

## Abstract

In eukaryotes, DNA is packaged into a basic unit, the nucleosome which consists of 147 bp of DNA wrapped around a histone octamer composed of two copies each of the histones H2A, H2B, H3 and H4. Nucleosome structures are diverse not only by histone variants, histone modifications, histone composition but also through accommodating different conformational states such as DNA breathing and dimer splitting. Variation in nucleosome structures allows it to perform a variety of cellular functions. Here, we identified a novel spontaneous conformational switching of nucleosomes under physiological conditions using single-molecule FRET. Using FRET probes placed at various positions on the nucleosomal DNA to monitor conformation of the nucleosome over a long period of time (30–60 min) at various ionic conditions, we identified conformational changes we refer to as nucleosome gaping. Gaping transitions are distinct from nucleosome breathing, sliding or tightening. Gaping modes switch along the direction normal to the DNA plane through about 5–10 angstroms and at minutes (1–10 min) time scale. This conformational transition, which has not been observed previously, may be potentially important for enzymatic reactions/transactions on nucleosomal substrate and the formation of multiple compression forms of chromatin fibers.

## INTRODUCTION

In eukaryotes, DNA is packaged into a basic unit, the nucleosome (1). Nucleosomes are regularly arranged at approximately every 200 base pairs along the DNA like ‘beads on a string’, separated by short DNA linker (2). Each nucleosome consists of 147 bps of DNA wrapped around histone octamer core composed of two copies each of the histones H2A, H2B, H3 and H4 (3). DNA is stably packed on histone surface by electrostatic interaction and hydrogen bonds between DNA and the protein core (3).

Nucleosome structures are diverse due to histone variants, histone modifications (2) and variations in its composition (4). Histone variants CenH3, H2A.Bdb and H2ALap1 form nucleosomes with loosely organized DNA termini (2). During transcription, replication and remodeling, parts of nucleosome such as H2A/H2B dimer dissociate creating additional structural intermediates and diversity (4–7). Even intact nucleosomes are highly dynamic and may deviate from the canonical crystal structure by DNA breathing (8–12), H2A/H2B dimer splitting (2,11,13,14) and nucleosome gaping (15,16). In DNA breathing and opening excursions, nucleosomal DNA ends unwrap from the histone core partially and reversibly on a rapid time scale (10–250 ms) (9,17). Spontaneous unwrapping of nucleosomal DNA ends makes chromatin more accessible to DNA binding factors (8,18). In dimer splitting, partial disruption of contacts between H2A/H2B dimers and histone (H3/H4)_2_ tetramer occurs while dimers maintain contact with DNA. In nucleosome gaping transition, two turns of nucleosomal DNA opens further apart relative to each other along the axis normal to the nucleosome plane. Among the three types of conformational transitions, only breathing and dimer splitting transitions have been observed experimentally. Nucleosome unwrapping occurs spontaneously in under physiological solution conditions (9,10,12). Nucleosome dimer splitting was observed only in elevated salt conditions (13). Nucleosome gaping was proposed theoretically in order to accommodate tightly package of chromatin higher order structure, the 30 nm fiber (15), but there has not been experimental demonstration.

FRET (Fluorescence Resonance Energy Transfer) method is based on the energy transfer between two fluorophores which depends on the distance between them (19,20). A donor fluorophore and an acceptor fluorophore can be attached to specific locations on nucleosomal DNA, thus allowing us to follow conformational changes resulting in changes in the donor-acceptor distance. FRET has been widely used to investigate conformational dynamics of bio-molecules in general and specifically for nucleosomes (4,9,12,13,21–24). Using stopped-flow FRET, fluorescence correlation spectroscopy and single-molecule FRET, it has been shown that spontaneous unwrapping of nucleosome takes place in the milliseconds time scale (the wrapped and unwrapped states last for ∼ 250 ms and ∼ 10–50 ms, respectively) (9,12,23).

Here we used single molecule FRET (smFRET) (20,25) to obtain evidence that, under physiological conditions, two turns of nucleosomal DNA can undergo slow spontaneous local switching transitions. This structural switching is along the direction perpendicular to the DNA plane, which we call gaping, a type of motion very different from the canonical in-plane motion previously observed. The amplitude of gaping is estimated to be about ∼5–10 angstroms and its time scale is minutes.

## MATERIALS AND METHODS

### Preparation of DNA constructs

dsDNA constructs of 181 base pairs (bps) were prepared by polymerase chain reaction (PCR) amplification of a plasmid which contains 147 bp 601 positioning sequence (Addgene Plasmid 26656: pGEM-3z/601 plasmid). On the DNA construct, the 601 sequence is flanked by a14 bp spacer to biotin and 20 bp spacer on the other side. The construct was tethered to a polymer-passivated surface via biotin. PCR primers were synthesized by Integrated DNA Technologies. Details of all primers were listed in our previous publication (26) and supporting information. An amino modification (5AmMC6T) at a designated location was placed on the primers for labeling with Cy3 (donor) or Cy5 (acceptor) as previously described (25).

### Nucleosome reconstitution

PCR-amplified 601 templates were reconstituted with *X. laevis* recombinant histone octamer (purchased from Colorado State University) by salt dialysis (20). Reconstituted nucleosomes were stored at 4^o^C in the dark typically at concentrations of 100–200 nM and used within 2 weeks. The efficiency of nucleosome reconstitution was measured by 5% native PAGE gel electrophoresis.

### Single-molecule FRET experiments

A microscope quartz slide was coated with polyethyleneglycol (PEG) (mixture of mPEG-SVA and Biotin-PEG-SVA, Laysan Bio) according to (25). The nucleosome sample was immobilized on the PEG-coated slide at 50 pM in nucleosome dilution buffer (10 mM Tris-HCl pH 8.0, 50 mM NaCl, 1 mM MgCl_2_) through a biotin/neutravidin linker. Single-molecule FRET data were taken in imaging buffer (50 mM Tris-HCl pH 8, 1 mM MgCl_2_, 0.5% w/v D-Glucose (Sigma), 165 U/ml glucose oxidase (Sigma), 2170 U/ml catalase (Roche), 3 mM Trolox (Sigma)) and a desired amount of NaCl) using a home-build prism-type total internal reflection fluorescence microscope (25). Image integration times were 500 ms for obtaining long single-molecule FRET traces and 50 ms for building FRET histogram.

### Single-molecule FRET data analysis

Single-molecule FRET data were analyzed using scripts written in IDL and Matlab. Briefly, time traces of individual molecule were extracted from movies recorded of 35 micron x 70 micron imaging area containing typically 500 molecules. We subtracted from the intensity time traces the background determined after photobleaching of both fluorophores. FRET value was calculated as the ratio between the acceptor intensity and the total intensity of the donor and acceptor after applying corrections for donor leakage into the acceptor channel (0.12) and gamma factor (1.7) (25). Mean dwell time of each FRET state was calculated by dividing the total dwell time in each state by the number of transitions leaving that state. If not indicated, sm FRET histograms were constructed by averaging FRET values of each molecule over 500 ms (10 frames of 50 ms in duration) so that a random 500 ms snap shot of a molecule contributes one count to the histogram, and then normalized to the total counts.

## RESULTS

### Nucleosome undergoes spontaneous conformational switching

**Figure 1.**
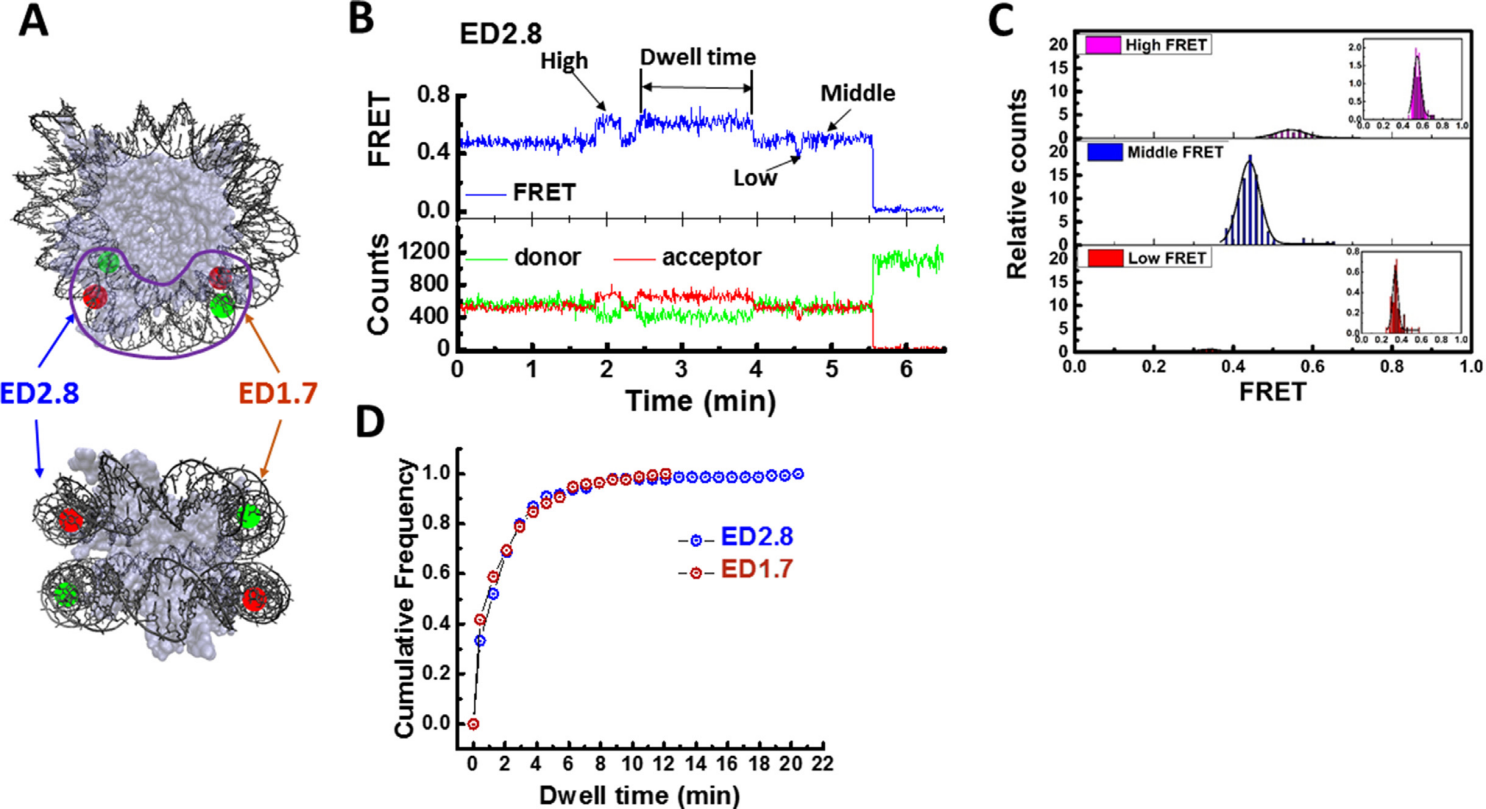
Slow spontaneous conformational switching of single nucleosome. (**A**) Fluorescent labeling scheme (front view and side view): Cy3 (green dot) and Cy5 (red dot) are attached on the two turns of the nucleosomal DNA (Crystal structure: 3MVD). Slow conformation switching occurs in the region marked by the purple line. (**B**) Single molecule time traces of donor intensity (green), acceptor intensity (red) and calculated FRET efficiency (blue) show spontaneous switching between three FRET levels. (**C**) Normalized FRET histograms of individual FRET states of ED2.8 construct. To build these histograms, we recorded FRET time traces of approximately 1000 molecules for 50 min with 0.5 s per frame. For each molecule, we extracted average of FRET at each state (high, middle and low FRET states) separately to build histogram of individual states. The total count of each histogram was normalized to its relative population (11.83%, 84.84% and 3.46% for high, middle and low FRET states, respectively). The insets of the top and the bottom panel are the room-in histograms of high and low FRET states. (**D**) Cumulative distribution function of the dwell time of the high FRET state at 50 mM NaCl for ED2.8 (number of dwells *N* = 249) and ED1.7 (*N* = 170) probes.

To get clearly interpretable smFRET signals, we chose to use the 601 sequence which positions nucleosome at a defined translation frame (27) and has been used in previous high resolution single molecule studies (5,6,13,18,26,28–38). A donor fluorophore (Cy3) was attached to the DNA inner turn that primarily contacts the histone tetramer, at the 45 nucleotide from the 5′ end of the bottom strand (or J strand), and the acceptor was attached to the DNA outer turn that primarily contacts the histone dimer, at the 27 nucleotide from the 5′ end of the top strand (or I strand) (Figure 1A). We named this labeling scheme ED2.8 (I27–J45). The DNA construct was generated by PCR amplification using a Cy3-labeled primer and a Cy5-labeled primer. One of the primers contains a biotin at the 5′ end for surface immobilization. In all our experiments, we used 1 mM Mg^2+^, which we found to increase the stability of the nucleosome, and specified concentration of NaCl. Reconstituted nucleosomes display a single broad high FRET peak in the smFRET efficiency histogram plus a low FRET peak attributed to the free DNA (Supplementary Figure S1). smFRET traces of ED2.8 in 50 mM NaCl solution revealed spontaneous transitions between three FRET states (Figure 1B and C): the high FRET state at 0.545 ± 0.003, the middle FRET state at 0.440 ± 0.001 and the low FRET state at 0.339 ± 0.003 (Figure 1C and Table 1, the errors represent standard errors of measurement). The nucleosome resided predominantly in the middle FRET state (84.7% of total observed time) and transiently stayed at higher (11.8%) and lower (3.5%) FRET states. Mean dwell time of each FRET state, calculated by dividing the total dwell time in each state by the number of transitions leaving that state, was 418.6 s, 100.25 s and 42.48 s for the middle, high and low FRET states, respectively (Table 2). This result implies that the nucleosome can undergo slow conformational switching.

**Table 1.** FRET values of three FRET states and their conversion to the distances between the donor and acceptor fluorophores of the ED2.8 construct

	FRET	Distance (nm)
High FRET	0.545 ± 0.003	5.7
Middle FRET	0.440 ± 0.001	6.1
Low FRET	0.339 ± 0.003	6.6

**Table 2.** Relative population and distribution and dwell time of each state probed by ED2.8 and ED1.7 constructs

	ED2.8		ED1.7	
	Mean dwell time (sec)	Relative population	Mean dwell time (sec)	Relative population
High FRET	100.25	11.83%	121 s	11.44%
Mid FRET	418.6	84.71%	569 s	80.16%
Low FRET	42.48	3.46%	102 s	8.4%

Next, we tested if the similar conformation dynamics can be observed at an equivalent site on the other side of the nucleosome. We designed a new DNA construct ED1.7 with the FRET pair attached to the site opposite to ED2.8. The donor was attached to the 46^th^ nt of the top strand and the acceptor was attached to the 24^th^ nt of the bottom strand (I46–J24). The ED1.7 construct showed similar transitions between three FRET states (Supplementary Figure S2). To quantify the similarity between ED1.7 and ED2.8, we compared the distributions and dwell times of corresponding states. Similar to ED2.8, ED1.7 resided predominantly in the middle FRET state (80.16%) for an average of 569 s (Table 2) and briefly in the high FRET state (11.44% for an average of 121 sec) and low FRET state (8.4% for an average of 102 sec). Because the dwell time in the middle FRET state is many minutes long, photobleaching makes it difficult to build dwell time histograms required for accurate comparison of transition kinetics. Therefore, we chose to analyze the dwell time distribution of the high FRET state only. Cumulative distributions of dwell time of the high FRET state were identical between ED1.7 and ED2.8 (Figure 1D), giving the lifetimes of 99 ± 11.8 s and 106 ± 4.8 s, respectively. The comparison between the two labeling configurations confirmed that the spontaneous conformational switching of nucleosome occurs on both sides of the nucleosome. Due to the similarity of the distributions and average dwell times of the two FRET probe, it is likely that the high FRET states in both constructs are identical states, and the same for the middle and low FRET states. The particular labeling positions employed suggest that the conformational changes occur around the junction between the DNA inner turn, which is in contact with the (H3/H4)_2_ tetramer, and the DNA outer turn, which is in contact with the two H2A/H2B dimers (Figure 1A).

### Salt-dependent kinetics

**Figure 2.**
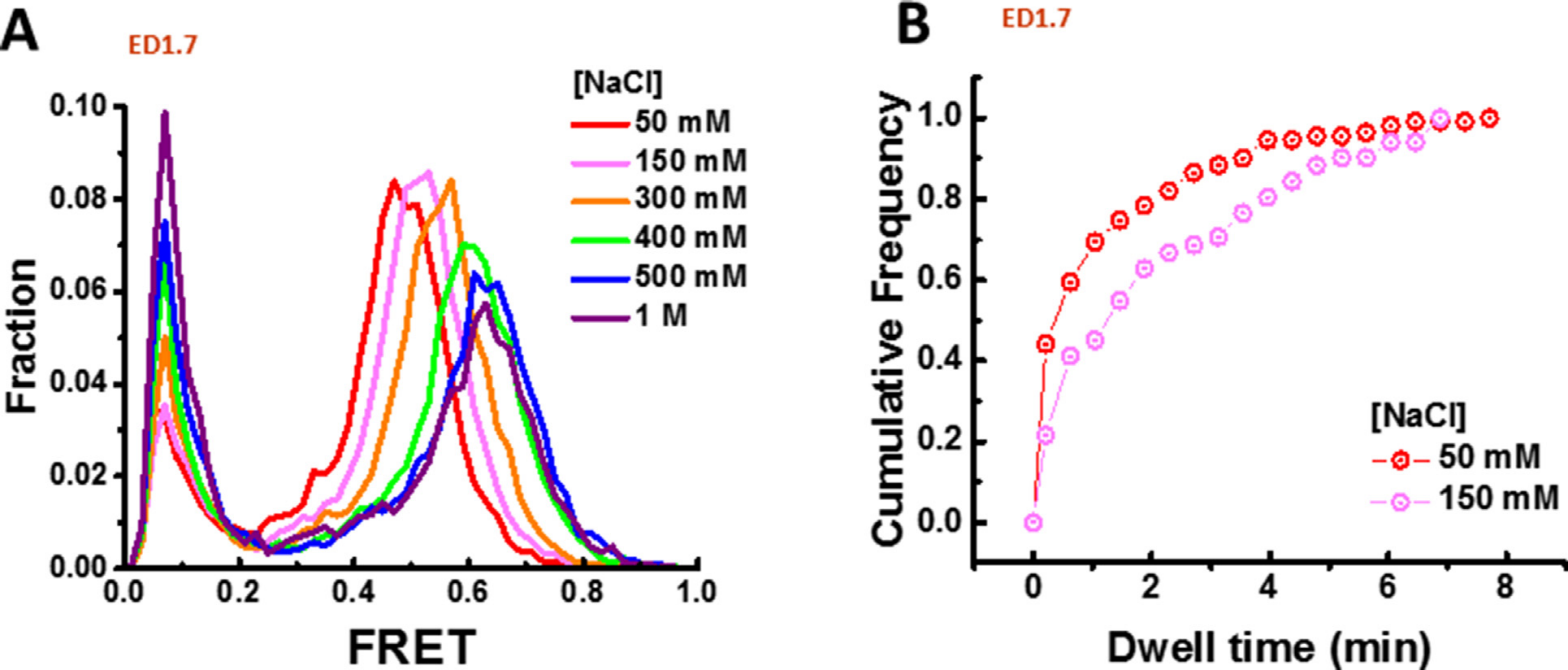
Salt-dependent kinetics. (**A**) FRET histogram of ED1.7 at different NaCl concentrations. (**B**) Cumulative distribution function of the dwell time of the high FRET state at 50 mM (number of dwells *N* = 170) and 150 mM (*N* = 140) NaCl.

Because the nucleosome stability is dependent on salt concentrations (13), we examined the conformational switching kinetics at different ionic conditions. smFRET histograms of the ED1.7 construct were recorded over a wide range of NaCl concentrations from 50 mM to 1 M. A fraction of nucleosomes disassembled and converted to free DNA at high salt concentrations, increasing the low FRET population (Figure 2), as previously reported (13). For the remaining nucleosomal peak, we observed progressive increases in the FRET value with increasing NaCl concentration (Figure 2A). This is in contrast to a FRET decrease observed with increasing salt when the labeling configuration is changed to report on the unraveling of the nucleosomal DNA ends, termed ED2–1 (26) (Supplementary Figure S3). In addition, the low FRET peak we attribute to free DNA did not change its position with NaCl concentration (Figure 2A). These comparisons rule out the possibility that the increase in FRET of the ED1.7 construct stems from a change in photophysical properties of the donor and acceptor caused by changing salt conditions because such an effect would also show FRET increases for the end labeling configuration or for the free DNA peak. Instead, the increase in FRET indicates a structural change induced by high ionic condition.

**Figure 3.**
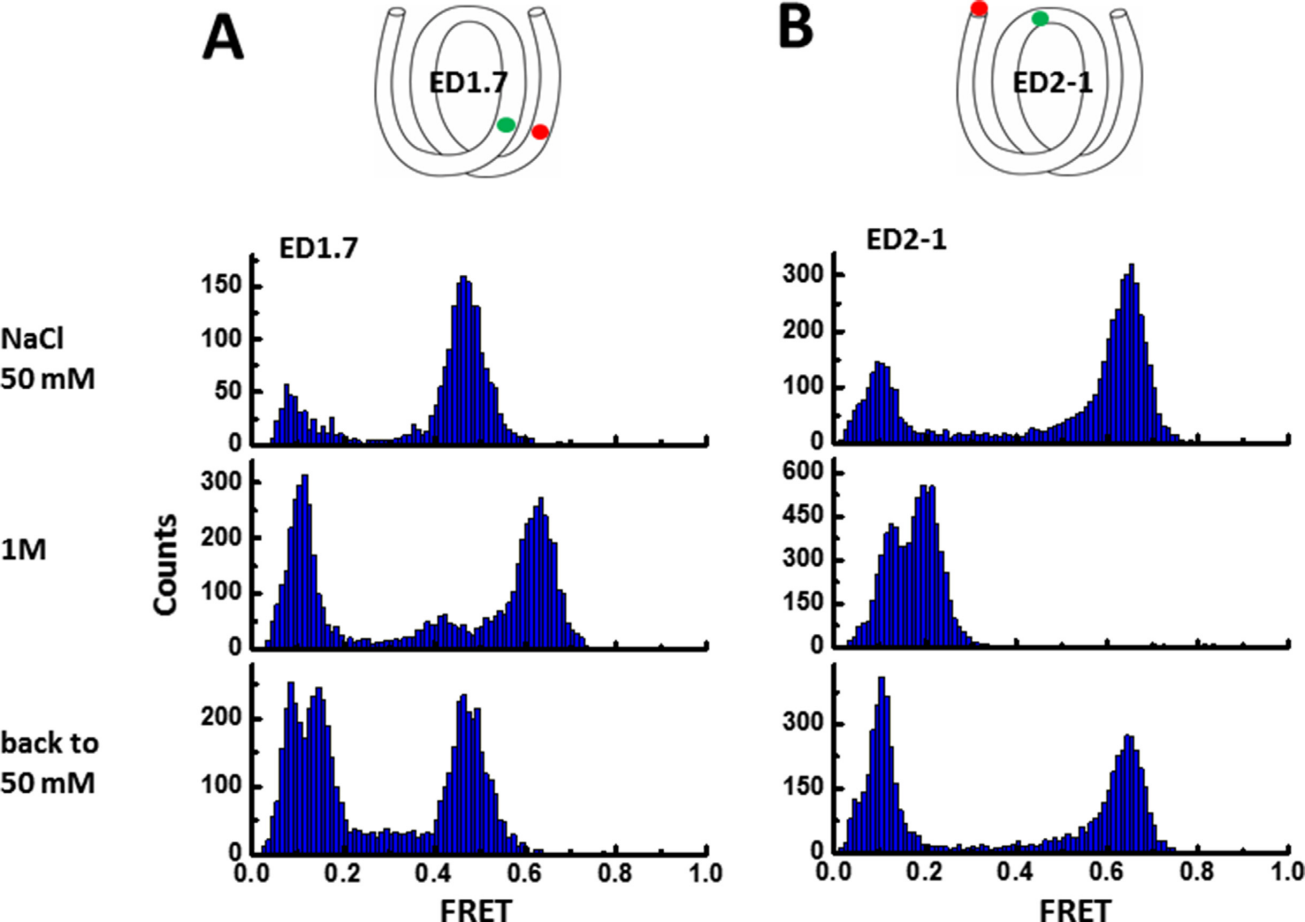
Checking nucleosome integrity at elevated salt concentration. FRET histogram of ED1.7 (**A**) and ED2–1 (**B**) at 50 mM NaCl, 1 M within 10 min of incubation and then back to 50 mM NaCl after 10 min incubation in 1 M NaCl. We found that a majority of the nucleosomes recover fully, as judged by FRET, after 10 min incubation 1 M NaCl, possibly because 1 mM MgCl_2_ which we have in all our solutions increases the nucleosome stability.

Next, we examined the possibility that the FRET increase may be due to loss of histone proteins from the DNA at elevated salt concentrations. If the FRET increase were due to histone dissociation at high salt, for example loss of H2.A/H2.B dimer(s), such an irreversible change would give a change in FRET before and after an excursion to high salt conditions. Instead, we found that the main nucleosomal FRET peak for ED1.7 remains in the same position after changing NaCl concentration from 50 mM to 1 M and then back to 50 mM (Figure 3A), making it unlikely that salt-induced FRET changes are due to histone dissociation. In addition, if the dimers on the nucleosome are lost in high salt, the DNA termini of the nucleosome may not be able to wrap back to the initial state upon returning the molecule to low salt. Instead, the main nucleosomal FRET peak for ED2–1 remained in the same position after changing NaCl concentration from 50 mM to 1 M and then back to 50 mM (Figure 3B), further suggesting that a majority of nucleosomes retain all histone proteins at 1 M NaCl. As an additional test, we performed bulk FRET experiment at 1M NaCl of the ED1.7 nuclesome and of ED1.7 DNA mixed with histone octamer without prior assembly. FRET of the nucleosome sample at 1M NaCl is significant higher than that of DNA mixed with histone octamer which is not expected to produce properly assembled nucleosomes (Supplementary Figure S2B). Therefore, this bulk FRET experiments also suggests that at 1M NaCl, a majority of molecules remain as a full nucleosome.

The increase in FRET of the ED1.7 probe could come from the shift of all three FRET sub-states or the change in their relative distribution, for example, from the middle FRET state to the high FRET state. To distinguish between these two possibilities, we compared the dwell time distributions of the high FRET state at the two different salt concentrations (Figure 2B). As the salt concentration increased, the cumulative dwell time distribution shifted to longer dwell times, suggesting that dwell time increase in the high FRET state contributes to the increases in the time-averaged FRET values induced by salt.

### Nucleosome gaping

**Figure 4.**
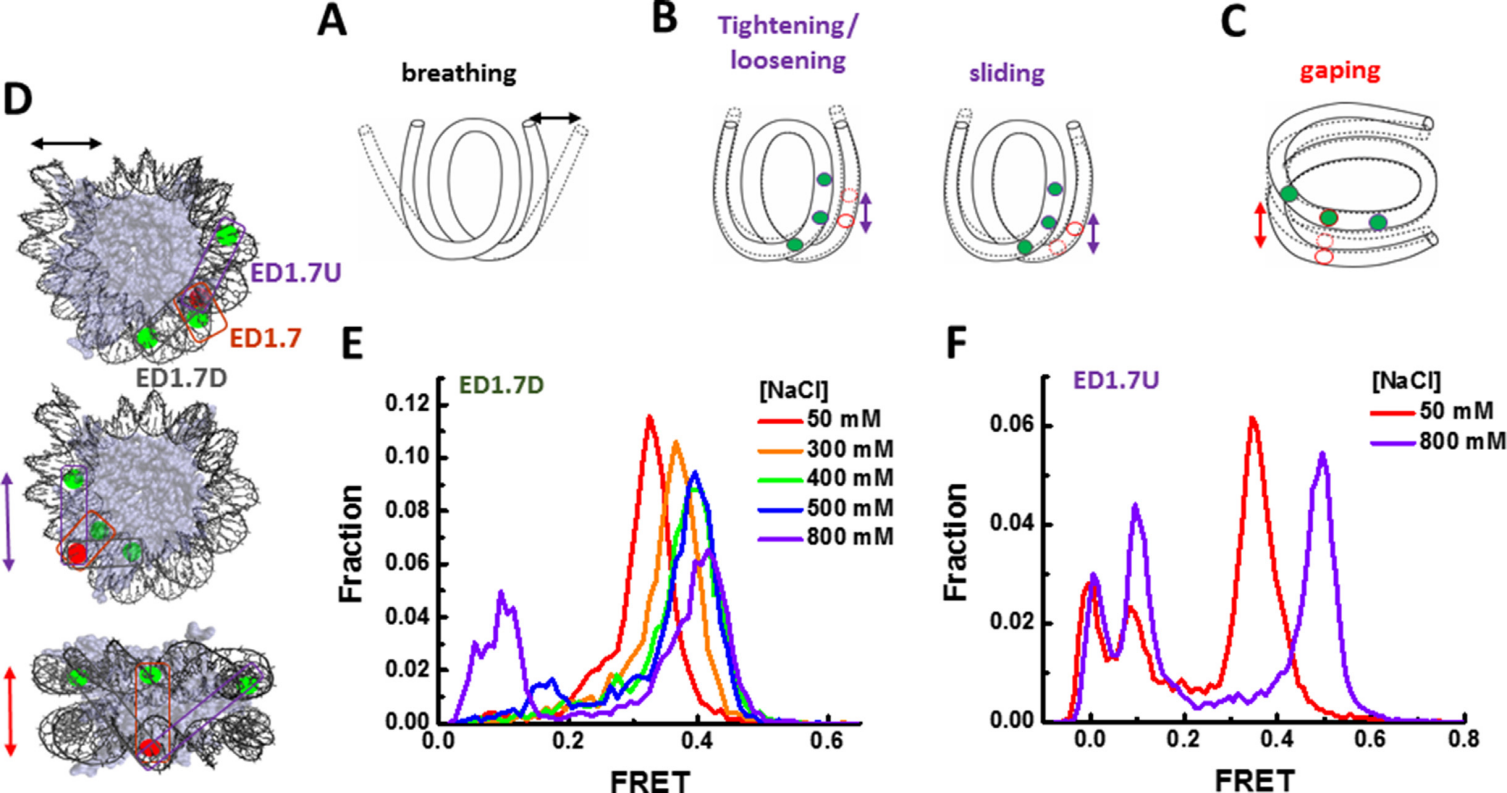
Nucleosome gaping. (**A**–**C**) Possible structural modes of nucleosome conformational dynamics: breathing (A), tightening/loosening and sliding (B), and gaping (C). (**D**) Illustration of three labeling schemes ED1.7, ED1.7U and ED1.7D overlaid on the nucleosome crystal structure. All three labeling schemes share the acceptor position at the 24 nucleotide from the right end (J24) of the 601 sequence on the bottom strand and the donors at labeled at 46, 57 and 38 nucleotides from the left end on the top strand (I46, I57 and I38), respectively. (**E** and **F**) FRET histogram of ED1.7D and ED1.7U at different NaCl concentrations.

Next, we investigated the molecular origin of the conformational switching. We considered four possible structural modes of conformational dynamics that could in principle result in the observed switching in FRET: DNA breathing, DNA tightening/loosening, nucleosome sliding, and nucleosome gaping. In DNA breathing (Figure 4A), the nucleosomal DNA ends transiently dissociate from the histone surface resulting in a relative change in distance between two nucleosomal DNA turns (8,9,12). In tightening/loosening (Figure 4B), DNA overwraps or underwraps around the histone octamer surface, decreasing or increasing the number of bp bound to the histone core, respectively (22). In sliding, the number of bp of DNA bound to the histone core is unchanged but DNA in contact with the octamer surface shifts upstream or downstream. In gaping, two turns of nucleosomal DNA moves relative to each other in the direction perpendicular to the two DNA planes (15). With both tightening/loosening and slide modes, the relative change in the two DNA turns is along the wrapping direction, i.e. within the DNA plane, while in the nucleosome gaping mode (Figure 4C) switching happens in a directional perpendicular to the DNA planes.

Stopped-flow FRET and fluorescence correlation spectroscopy measurements (9) and smFRET measurements (12) determined the rates of spontaneous nucleosome unwrapping and rewrapping in the breathing mode to be in the millisecond time scales (10–250 ms) which is two orders of magnitude faster than the switching that we observed here (1–10 min). Moreover, in the breathing mode, unwrapping of nucleosomal DNA ends from the histone core is facilitated by high salt (13), resulting in a decrease in time-averaged FRET as salt increases (Supplementary Figure S3, Figure 3B). Here the observed switching results in FRET increase with increasing NaCl concentration. Combining the difference in transition rates and the trend of FRET change with salt titration, we ruled out DNA breathing as the source of observed FRET switching.

To distinguish between tightening/loosening, sliding and gaping, we designed two additional labeling schemes ED1.7U and ED1.7D which share the acceptor position on the outer DNA with the ED1.7 scheme (Figure 4D). The two donors in ED1.7U and ED1.7D were located at the two opposite positions compared to the donor in ED1.7. In tightening/loosing or sliding, the outer DNA turn will move relative to the inner DNA turn such that if the acceptor on the outer turn get closer to the donor of ED1.7D on the inner turn, it will be further away from the donor of ED1.7U which is on the opposite side, and vice versa. Therefore, the sign of FRET change of ED1.7U should be opposite to that of ED1.7D. However, single molecule FRET histograms of both ED1.7D and ED1.7U (Figure 4E and F) showed FRET increases with increasing NaCl as was observed for ED1.7 (Figure 2A). This ruled out that tightening/loosening or sliding as the source of conformational switching. Instead, our data support the gaping model because gaping allows an increase in FRET of all three pairs ED1.7. ED1.7U and ED1.7D as the two DNA planes move closer or farther apart like in a clam shell (Figure 4C).

## DISCUSSION

Diverse nucleosome compositions involving histone variants and histone modifications accommodate specific tasks in gene regulation. Nucleosomes composed of the same DNA and protein components also display conformational dynamics such as breathing and opening of tetramer/dimer interface. Spontaneous breathing of nucleosomal DNA ends make nucleosome substrates accessible to transcription factors, polymerases and chromatin remodelers.

Here, we discovered that nucleosomes can undergo a local switching motion along the direction perpendicular to the DNA plane, a type of motion very different from the canonical in-plane motion previously observed. We call this switching as gaping similar to the direction of molecular conformation change proposed theoretically by Mozziconacci and Victor (15). They proposed that cooperative gaping transitions of neighboring nucleosomes may contribute to the compaction and decompaction of the chromatin fiber. Our results indicate that a free nucleosome in solution can adopt and switch between multiple configurations which may dictate the heterogeneous enzymatic reactions on chromatin substrates and the formation of multiple compression forms and functional states of chromatin structures (15,39).

SmFRET with resolution of sub-nanometer allowed us to discover a new conformation switching of free nucleosome under physiological conditions. To minimize the effect of labeling, we incorporated fluorophores to DNA through a C6 linker, which may increase rotational mobility of the fluorophores. We used the kappa square factor of 2/3 to estimate *R*_0_ and absolute distances below, which would be valid strictly only if the fluorophores’ orientation is fully averaged out within the fluorescence lifetimes. Therefore, our absolute distance estimation is only approximate. The distance between the donor and acceptor in the three FRET states are 5.7 ± 0.01 nm (high FRET state), 6.1 ± 0.004 nm (middle FRET state) and 6.6 ± 0.015 nm (low FRET state) (Table 1, conversion of FRET to distance using the characteristic Förster distance *R*_0_ = 5.9 nm (40)), while the distance between the two nucleotides which carry the fluorophores shown in the crystal structure (pdb file 3MVD)(41) is 3.3 nm. If we allow the extension in the distance of the two fluorophores caused by the linker to be 2.2 nm (42), the high FRET state would correspond to the structure found in the crystal structure (41). However, the configuration of the C6 liker is unknown, therefore, we are not able to identify which of the three states corresponds to the canonical nucleosome structure found in the crystallographically determined structures. Nevertheless, we can estimate the relative distance changes in gaping transitions to be approximately 0.5–1 nm. This comparison between different states of the gaping transition does not require absolute distance information and therefore the qualitative conclusion of the gaping transitions is independent of the assumption that the fluorophores are freely rotating. Within the scope of this study, we have no information on possible conformational changes of the histone octamer itself. As a result, we could not probe for dimer splitting which may or may not accompany gaping transitions. Further studies with approaches such as precision FRET (21) or small-angle X-ray scattering (43) are needed to resolve the detailed structural changes associated with nucleosome gaping.

## SUPPLEMENTARY DATA

Supplementary Data are available at NAR Online.

SUPPLEMENTARY DATA
