# Supplementary material for: Nucleosomes undergo slow spontaneous gaping
Source: Nucleic Acids Res. 2015 Mar 30;43(8):3964–71. doi: 10.1093/nar/gkv276 (PMC4417179; doi:10.1093/nar/gkv276)
Supplement: SUPPLEMENTARY DATA [file supp_43_8_3964__index.html]

Nucleosomes undergo slow spontaneous gaping — SUPPLEMENTARY DATA 

# Nucleosomes undergo slow spontaneous gaping

## SUPPLEMENTARY DATA

**Files in this Data Supplement:**

- SUPPLEMENTARY DATA
