# Supplementary material for: Nucleosomes undergo slow spontaneous gaping
Source: Nucleic Acids Res. 2015 Mar 30;43(8):3964–71. doi: 10.1093/nar/gkv276 (PMC4417179; doi:10.1093/nar/gkv276)

Supporting information

Nucleosomes Undergo Slow Spontaneous Gaping

Thuy T. M. Ngo[1] and Taekjip Ha[1,2,3,4*]

[1] Center for Biophysics and Computational Biology, University of Illinois at Urbana-Champaign, Urbana, IL 61801-2902, USA.

[2]Department of Physics, Center for Physics in Living Cells, University of Illinois at Urbana-Champaign, Urbana, IL 61801-2902, USA.

[3]Institute for Genomic Biology, University of Illinois at Urbana-Champaign, Urbana, IL 61801-2902, USA.

[4]Howard Hughes Medical Institute, University of Illinois, Urbana, IL 61801-2902, USA.

*To whom correspondence should be addressed. Tel: +1.217.265.0717; Fax: +1.217.244.7187; Email: tjha@illinois.edu

List of primers:

I27:

5’ GG GCGGCGACCT /idSp/GGACCCTATA CGCGGCCGCC CTGGAGAATC CCGGTGCCGA GGCCGCTCAA TTGGTCG/iAmMC6T/AG A

J45:

5'- /5Biosg/TGTT CAATACATGC ACAGGAT GTATATATCT GACACGTGCC TGGAGACTAG GGAGTAA/iAmMC6T/CC C

I46:

5’-/5Biosg/TATA CGCGGCCGCC CTGGAGAATC CCGGTGCCGA GGCCGCTCAA TTGGTCGTAG ACAGC/iAmMC6T/CTA

J24:

5'- GG GCGGCGACCT /idSp/GGTCGCTGTT CAATACATGC ACAGGAT GTATATATCT GACACG/iAmMC6T/GCC TGGA

I57:

5’-/5Biosg/TATA CGCGGCCGCC CTGGAGAATC CCGGTGCCGA GGCCGCTCAA TTGGTCGTAG ACAGCTCTAG CACCGC/iAmMC6T/TAA

I38:

5’-/5Biosg/TATA CGCGGCCGCC CTGGAGAATC CCGGTGCCGA GGCCGCTCAA TTGGTCG/iAmMC6T/AG A

I68:

5’-/5Biosg/TATA CGCGGCCGCC CTGGAGAATC CCGGTGCCGA GGCCGCTCAA TTGGTCGTAG ACAGCTCTAG CACCGCTTAA ACGCACG/iAmMC6T/AC G

J7:

5'- GG GCGGCGACCT /idSp/GGTCGCTGTT CAATACATGC ACAGGA/iAmMC6T/ GTATATA

I-1:

5’-/5Biosg/TATA CGCGGCCGC/iAmMC6T/ CTGGAGAA/iAmMC6T/C CCGGT

J79:

5'- GG GCGGCGACCT /idSp/GGTCGCTGTT CAATACATGC ACAGGAT GTATATATCT GACACGTGCC TGGAGACTAG GGAGTAATCC CCTTGGCGGT TAAAACGCGG GGGACAGCGC G/iAmMC6T/AC G

List of labeling schemes (Labeled positions are underlined**):**

**ED1.7 (I46,J24)**	CTGGAGAATC CCGGTGCCGA GGCCGCTCAA TTGGTCGTAG ACAGCTCTAG CACCGCTTAA ACGCACGTAC GCGCTGTCCC CCGCGTTTTA ACCGCCAAGG GGATTACTCC CTAGTCTCCA GGCACGTGTC AGATATATAC ATCCTGT
**ED2.8 (I27,J45)**	CTGGAGAATC CCGGTGCCGA GGCCGCTCAA TTGGTCGTAG ACAGCTCTAG CACCGCTTAA ACGCACGTAC GCGCTGTCCC CCGCGTTTTA ACCGCCAAGG GGATTACTCC CTAGTCTCCA GGCACGTGTC AGATATATAC ATCCTGT
**ED1.7U (I57,J24)**	CTGGAGAATC CCGGTGCCGA GGCCGCTCAA TTGGTCGTAG ACAGCTCTAG CACCGCTTAA ACGCACGTAC GCGCTGTCCC CCGCGTTTTA ACCGCCAAGG GGATTACTCC CTAGTCTCCA GGCACGTGTC AGATATATAC ATCCTGT
**ED1.7D (I38,J24)**	CTGGAGAATC CCGGTGCCGA GGCCGCTCAA TTGGTCGTAG ACAGCTCTAG CACCGCTTAA ACGCACGTAC GCGCTGTCCC CCGCGTTTTA ACCGCCAAGG GGATTACTCC CTAGTCTCCA GGCACGTGTC AGATATATAC ATCCTGT
**ED1 (I68,J7)**	CTGGAGAATC CCGGTGCCGA GGCCGCTCAA TTGGTCGTAG ACAGCTCTAG CACCGCTTAA ACGCACGTAC GCGCTGTCCC CCGCGTTTTA ACCGCCAAGG GGATTACTCC CTAGTCTCCA GGCACGTGTC AGATATATAC ATCCTGT
**ED2-1 (I-1,J79)**	T CTGGAGAATC CCGGTGCCGA GGCCGCTCAA TTGGTCGTAG ACAGCTCTAG CACCGCTTAA ACGCACGTAC GCGCTGTCCC CCGCGTTTTA ACCGCCAAGG GGATTACTCC CTAGTCTCCA GGCACGTGTC AGATATATAC ATCCTGT

Supplementary figures:

Figure S1: **Formation of nucleosomes with ED2.8, ED1.7. ED1.7D. ED1.7U labeling schemes**
(**A**) Nucleosomes with ED2.8, ED1.7. ED1.7D. ED1.7U labeling schemes migrate at identical positions on 5% Native PAGE.
(**B**): FRET histogram of these 4 nucleosomes

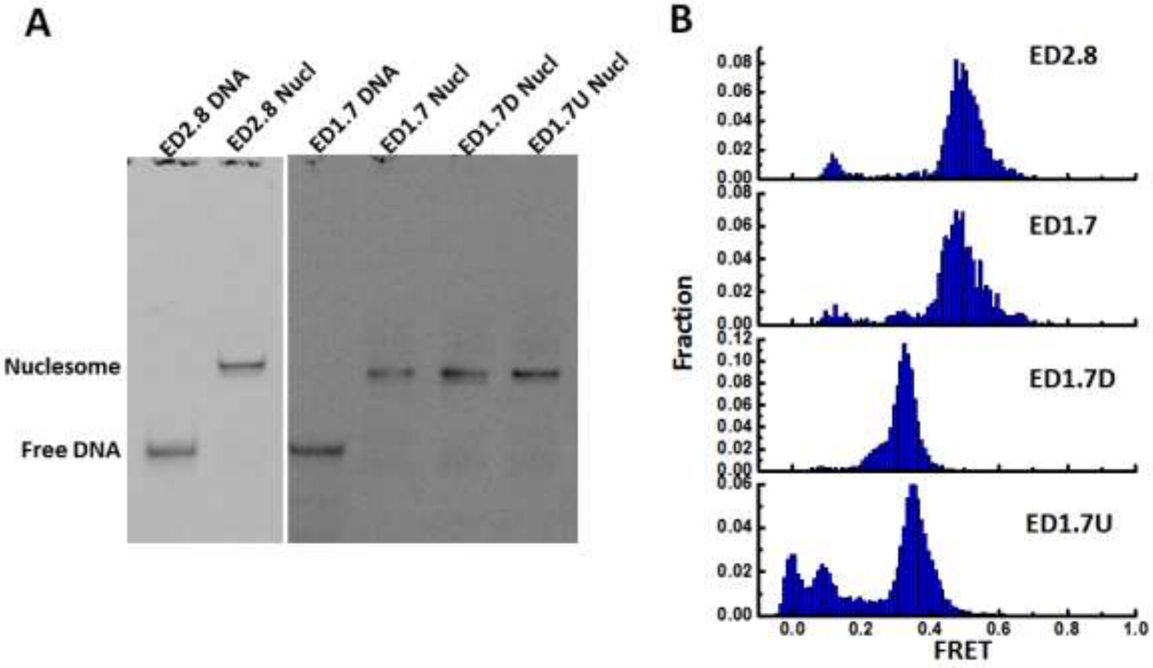

Figure S2: **FRET of the ED1.7 nucleosome**
(**A**) Single molecule time traces of donor intensity (green), acceptor intensity (red) and calculated FRET (blue) show spontaneous switching between three FRET levels indicating the coexistence of multiple states of the ED1.7 nucleosome.
(**B**) Bulk FRET of ED1.7 Nucleosome (black) vs. DNA and histone octamer added together (without prior assembly) (red) at 1 M NaCl, measured within 10 minutes after mixing in the final buffer (Tris-HCl pH 8 and 1 M NaCl).

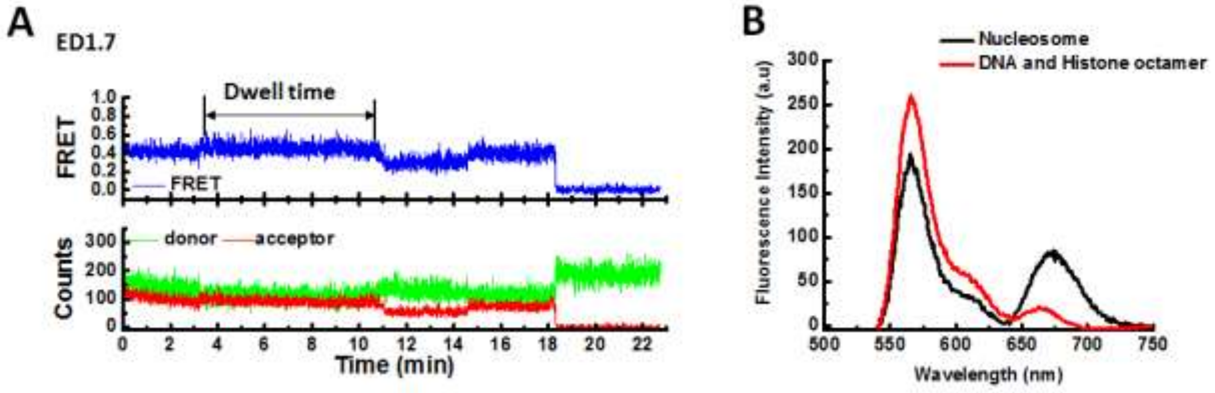

Figure S3: FRET histogram of ED1 (I68, J7) (**A, B**) nucleosome and ED2-1 (I-1, J79) (A, C) nucleosome at different NaCl concentrations showing a reduction in FRET as NaCl concentration increases.

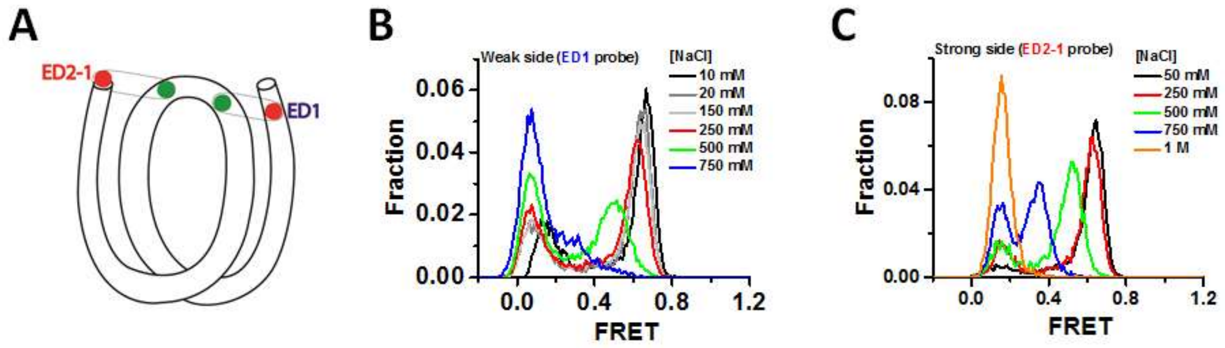

Supplement: SUPPLEMENTARY DATA [file supp_gkv276_nar-03693-m-2014-File006.pdf]
